# Urinary marker panels for aggressive prostate cancer detection

**DOI:** 10.1038/s41598-022-19134-3

**Published:** 2022-09-01

**Authors:** Tung-Shing Mamie Lih, Mingming Dong, Leslie Mangold, Alan Partin, Hui Zhang

**Affiliations:** 1 Department of Pathology, School of Medicine, Johns Hopkins University, Baltimore, MD 21231 USA; 2 The Brady Urological Institute, The Johns Hopkins School of Medicine, Baltimore, MD 21287 USA

**Keywords:** Cancer, Urological cancer, Prostate cancer

## Abstract

Majority of patients with indolent prostate cancer (PCa) can be managed with active surveillance. Therefore, finding biomarkers for classifying patients between indolent and aggressive PCa is essential. In this study, we investigated urinary marker panels composed of urinary glycopeptides and/or urinary prostate-specific antigen (PSA) for their clinical utility in distinguishing non-aggressive (Grade Group 1) from aggressive (Grade Group ≥ 2) PCa. Urinary glycopeptides acquired via data-independent acquisition mass spectrometry (DIA-MS) were quantitatively analyzed, where prostatic acid phosphatase (ACPP), clusterin (CLU), alpha-1-acid glycoprotein 1 (ORM1), and CD antigen 97 (CD97) were selected to be evaluated in various combinations with and without urinary PSA. Targeted parallel reaction monitoring (PRM) assays of the glycopeptides from urinary ACPP and CLU were investigated along with urinary PSA for the ability of aggressive PCa detection. The multi-urinary marker panels, combined via logistic regression, were statistically evaluated using bootstrap resampling and validated by an independent cohort. Majority of the multi-urinary marker panels (e.g., a panel consisted of ACPP, CLU, and Urinary PSA) achieved area under the curve (AUC) ranged from 0.70 to 0.85. Thus, multi-marker panels investigated in this study showed clinically meaningful results on aggressive PCa detection to separate Grade Group 1 from Grade Group 2 and above warranting further evaluation in clinical setting in future.

## Introduction

Screening of prostate cancer (PCa) has resulted in early intervention with decreased mortality and improved treatment outcomes^1–3^. However, in the past decades, PCa screening has led to an increase in the number of patients diagnosed^4,5^. Even though, the majority of patients with low-risk PCa can be safely managed on active surveillance^6–8^, many such patients still elect to undergo therapies (e.g., surgery or radiation)^9^. There are multiple factors to influence the patient’s decision on receiving an invasive treatment. First, clinical risk assessment tools for PCa before surgery primarily rely on prostate-specific antigen (PSA) level detected in blood test followed by prostate biopsy^10^. However, the blood PSA test is imprecise for distinguishing aggressive from latent or non-aggressive PCa, especially at the PSA level lower than 10 ng/mL^11–13^. Moreover, the invasiveness of biopsy test can cause pain and complications to patients, while the possibilities of sampling error and inter-observer grading inconsistency^10^. Second, although identifying the morphological differences between Gleason pattern ≤ 3 (lower aggressiveness) and Gleason pattern ≥ 4 (higher aggressiveness) is feasible, the specific molecular basis associated with the biological aggressive PCa is not fully understood^14^. As a result, PCa patients can be misclassified as at low-risk by the biopsy and later to be found with aggressive pathology at radical prostatectomy^14^. Finding biomarkers that are feasible to classify patients based on the feature differences between low-risk and high-risk PCa, which have not been captured by pre-treatment tests, would inspire greater confidence in decisions about pursuing active surveillance, or perhaps even offer new noninvasive methods to monitor patients on active surveillance.

Urine is an appealing source for finding noninvasive biomarkers. Since the urinary system is at proximity to the prostate, PCa-related substances (e.g., DNA, RNA, proteins) may be shed into urine. Moreover, the cancer-specific proteins are mixed with proteins secreted from other tissues in blood; thus, the interference from the background lowers the detection ability for PCa-specific proteins. On the other hand, urine has little exposure to other organs. Urine samples of PCa patients are expected to provide higher sensitivity and specificity in detecting proteins released from PCa^5,11,15,16^. Therefore, urine analysis for PCa may be more valuable than blood analysis. The majority of cell surface proteins or secreted proteins are known to be glycoproteins. Glycoproteins play essential roles in cancer development or progression and most of the FDA-approved biomarkers for cancer diagnosis and monitoring are glycoproteins^17^. Urine is rich in glycoproteins derived from urogenital system and glycoproteomic methods have been used to efficiently enrich and analyze glycoproteins from human urine^16,18,19^. Therefore, urine provides a solid basis for identifying urinary glyco-biomarkers associated with aggressive PCa^16,18^.

However, urine poses a great challenge for a high-throughput quantitative proteomic and glycoproteomic analysis due to low and variation in protein contents among individuals. The presence of interfering contents in urine can hamper the protease digestion during sample preparation for mass spectrometry-based analysis^15,20^. Therefore, urine samples are generally concentrated and the interfering substances are removed by filtration, washing, or precipitation^16,21^. To facilitate urine sample preparation, our laboratory developed a robot-based approach to automate tryptic digestion and isolation of glycopeptides from urine specimens^22^. This method allowed for processing large-scale urine specimens and the derived glycopeptides were subjected to data-independent acquisition mass spectrometry (DIA-MS) for high-throughput quantitative analysis with high reproducibility.

We previously discovered 20 glycopeptides from 20 glycoproteins using 142 urine specimens of PCa patients with Grade Group 1 (GG1, Gleason score = 6) and ≥ Grade Group 4 (GG4, Gleason score = 8; GG5, Gleason scores = 9–10)^13^. In this study, we first examined urinary marker panels composed of urinary PSA with/without one or more glycopeptides from four glycoproteins (ACPP, CLU, ORM1, and CD97) using the aforementioned 142 samples, which were not investigated in our previous published work. Since aggressive PCa includes Grade Groups 2 and 3 (GG2 and GG3; Gleason scores = 3 + 4 and 4 + 3) in real clinical setting; therefore, we further evaluated the urinary marker panels using an independent cohort contained urine samples with GG1, GG2, GG3, and GG4 to determine whether the urinary marker panels were also feasible for detecting aggressive PCa when GG2 and GG3 were included in the aggressive group.

## Methods

### Urine specimens

The first cohort (i.e., Cohort 1) containing post-digital rectal examination (DRE) urine samples from 75 aggressive PCa patients (Grade Group ≥ 4) and 70 non-aggressive PCa patients (GG1) were analyzed via quantitative analysis of DIA-MS. Three samples were excluded for the downstream analysis because low data quality. Therefore, 74 aggressive with Grade Group ≥ 4 and 68 non-aggressive with GG1 remained as training set for the downstream analysis. Parallel reaction monitoring (PRM) assays were developed and analyzed using the urine samples from the first cohort. A second cohort, independent from the first cohort, consisted of 156 post-DRE urine samples that were used for validation (Cohort 2). All the urine specimens were collected by the Department of Urology at Johns Hopkins University School of Medicine with approval from the Institutional Review Board of Johns Hopkins University that informed consent was obtained from all subjects/participants involved in the study. The clinical ELISA PSA assay (Access 2 Hybritech PSA Assay) was utilized to measure the total urinary PSA for each urine sample. Information of the clinical urine specimens of both cohorts is summarized in Tables S1 and S2.

### Experimental workflow

**Figure 1:**
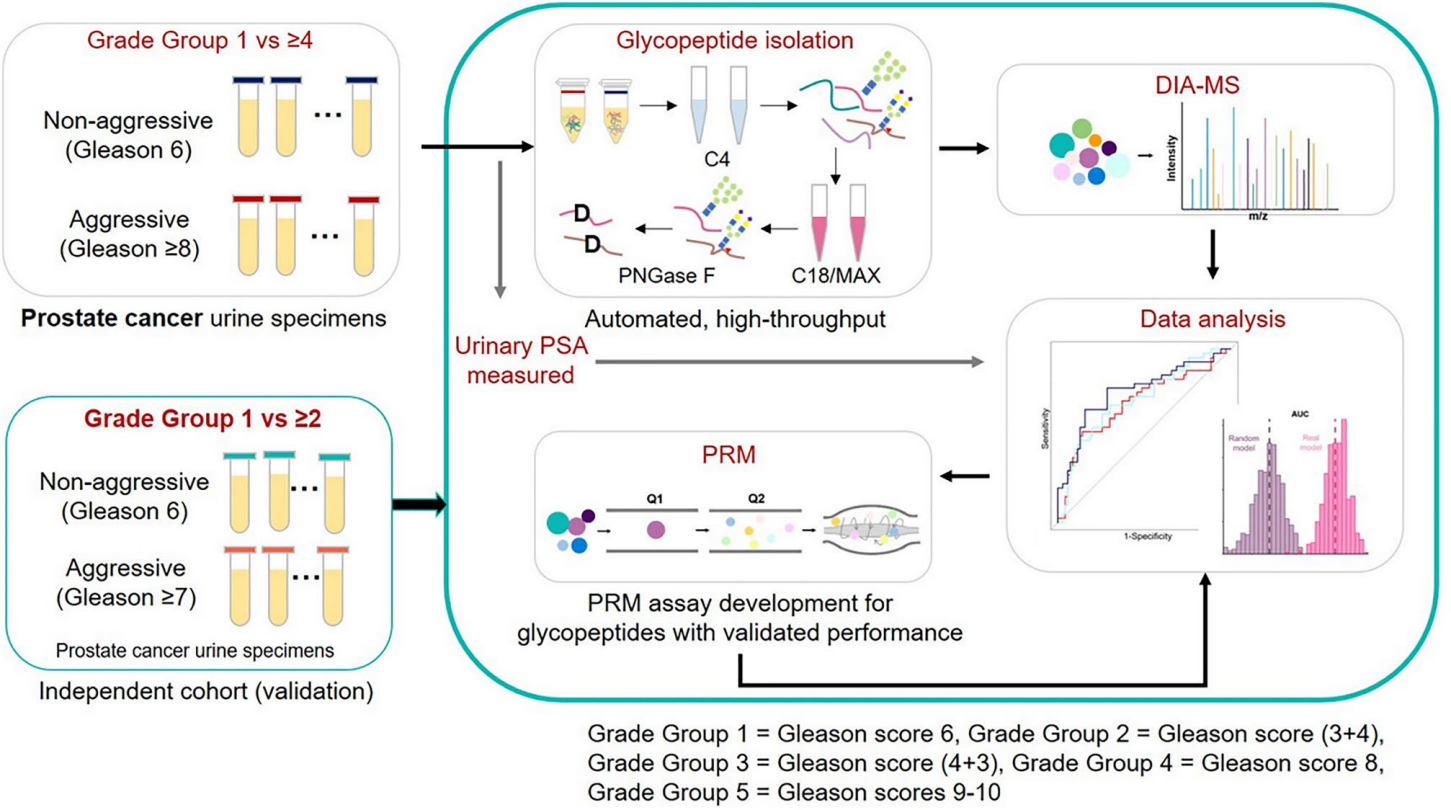
Experimental workflow of the study.

We utilized DIA-MS to quantitatively analyzed urinary glycopeptides which were acquired from 298 urine specimens, where 142 samples composed of only GG1 and ≥ GG4 for the training and 156 samples consisted GG1 to GG4 for the validation. The overall experimental workflow is illustrated in Fig. 1. Briefly, the urine samples (500 µL) were protease digested followed by intact glycopeptide enrichment using Versette (Thermo Scientific, Waltham, MA), which allowed automated high-throughput sample preparation as established by our group^20,22^. After removing N-glycans using PNGase F, N-linked glycosite-containing peptides (one tenth of the total glycopeptides enriched from 500 µL urine) and spike-in index retention time (iRT) peptides were subjected to DIA-MS analysis on Q-Exactive HF-X mass spectrometer. Additionally, the PRM assays were developed for glycopeptides with consistent performance based on DIA-MS data from Cohort 1 and the validation cohort. To quantitatively analyze glycopeptides, DIA raw data files were searched against the spectral library constructed using DDA data of pooled urine samples to identify and quantify glycopeptides via Spectronaut. The glycopeptides were normalized to the total protein amount for each urine sample prior to downstream analysis. For PRM assays, PRM raw files were analyzed by Skyline (version 20.1.0.76) and a minimum of four transitions was required for the correct detection of the target peptides. The ratios of endogenous glycopeptides (Light) to the corresponding heavy isotope-labeled internal standards (Heavy) for each urine sample were exported for downstream analysis. Detailed information on chemicals and reagents, automated sample preparation, enrichment of urinary glycopeptide, LC–MS/MS analysis, spectral library construction, PRM assay development can be found in our previous publications^13,23^.

### Statistical analysis

For each urinary marker panel (either composed of one candidate marker or multiple candidate markers), its discriminatory power through logistic regression was evaluated using receiver operating characteristic (ROC) analysis. The candidate marker data (missing values were median imputed) were log-transformed followed by z-score prior to ROC analysis. To ensure statistical stability of the results, we used bootstrap resampling (n = 500) of the data to construct and evaluate the predictive model of a urinary marker panel. The mean ROC curves were depicted based on bootstrap resampling results and an area under the curve (AUC) was computed for the mean ROC curve. The predictive models were further investigated using the independent validation cohort.

All the analyses were carried out in R (version 3.5). The predictive models were built using caret (version 6.0–85) and ROC curves were generated using pROC (version 1.13).

## Results

### Overview of the study

In the current study, we analyzed a urine cohort (Cohort 1 composed of 68 non-aggressive with GG1 and 74 aggressive with Grade Group ≥ 4; Table S1) which was studied in our recent published work^13^ by focusing on urinary PSA and potential multi-marker panels that were not explored previously via quantitative analysis of DIA-MS. To further evaluate the predictive models built from the first cohort, we utilized an independent post-DRE urine sample cohort, which contained 156 urine specimens (Cohort 2 as validation cohort composed of GG1 to GG4 urine samples; Table S2). Additionally, we have established PRM assays for the urinary glycopeptides from ACPP and CLU^23^. Therefore, we also re-analyzed the PRM data of the first cohort and further investigated using the validation cohort in this study. Figure 1 demonstrates the experimental workflow of the study as described in Methods. The quantitative data matrices used for the analyses are in Table S3. All methods were carried out in accordance with relevant guidelines and regulations.

### Performance of urinary marker panels based on quantitative analysis of DIA-MS

In our previous published work^13^, we discovered several urinary glycopeptides, for instance, FLN*ESYK from ACPP (where * indicated the glycosylation site) and EDALN*ETR from CLU, as candidate markers for detecting aggressive PCa which can serve as adjuncts to serum PSA test. We also established PRM assays for the aforementioned glycopeptides^23^. However, the potential predictive power of urinary PSA towards aggressive PCa detection as individual urinary candidate marker and in combination of other urinary glycopeptides was not analyzed previously. Thus, in the current study, we evaluated four urinary glycopeptides from ACPP, CLU, ORM1, and CD97 since they demonstrated good performance in our previous work, in combination with and without urinary PSA.

**Figure 2:**
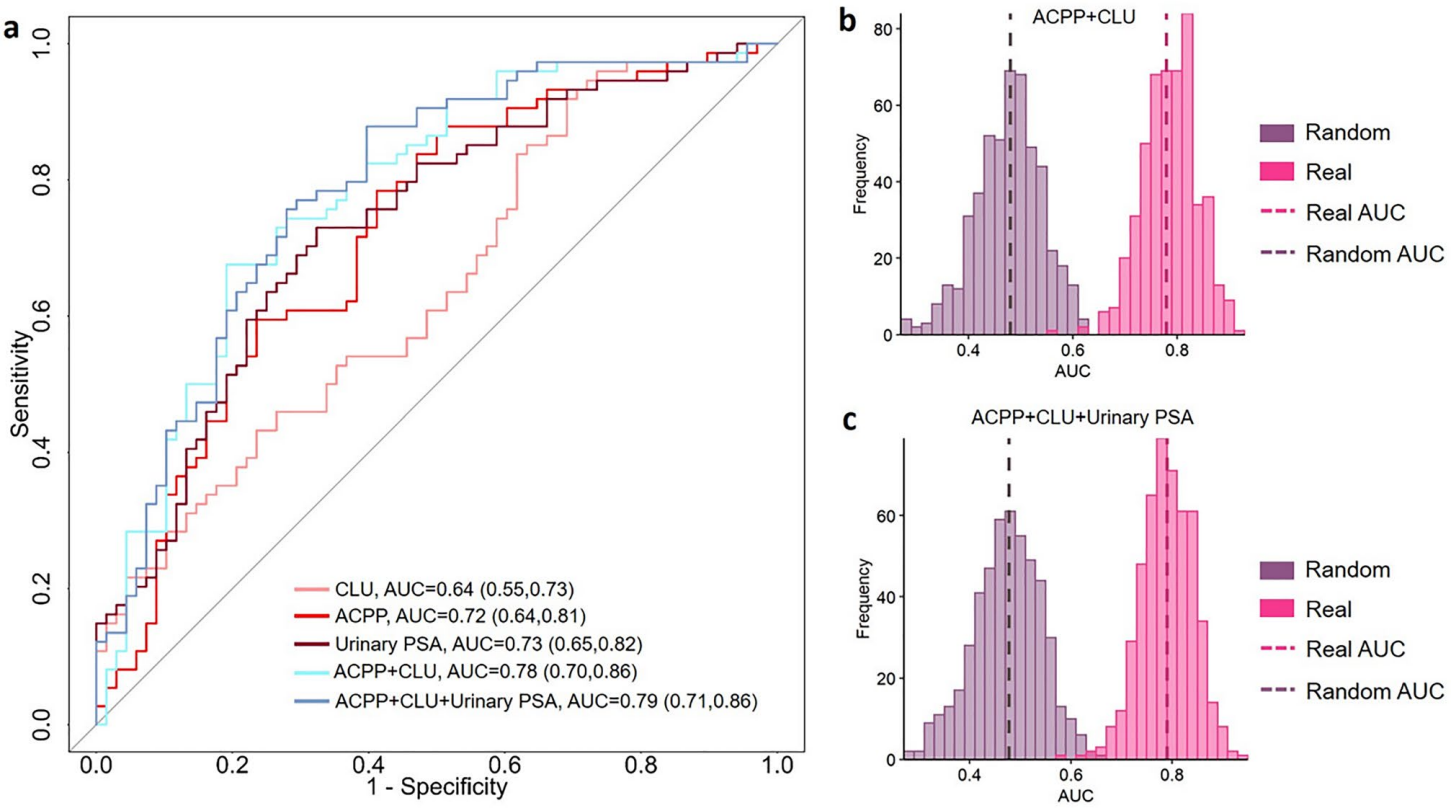
Urinary marker panels composed of ACPP, CLU (using quantitative analysis of DIA-MS), and/or urinary PSA. (**a**) ROC analysis of CLU, ACPP, urinary PSA, and multi-signature panels. (**b**) and (**c**) Comparison between real models and randomly generated models from label permutation based on bootstrap resampling. For simplicity, only protein names are used.

As shown in Fig. 2a (only protein names are labeled for simplicity), ACPP (AUC = 0.72) and urinary PSA (AUC = 0.73) have better performance compared to CLU for differentiating aggressive and non-aggressive PCa. However, when combining ACPP and CLU into a panel as well as combining all three candidate markers into another panel, we observed an improvement in aggressive PCa detection, where AUCs of 0.78 and 0.79 were obtained, respectively (Table S4). To ensure the results were reliable, we generated random models by label permutated the original data prior to bootstrap resampling (n = 500). By plotting the bootstrapping AUCs from both random models and the predictive models (i.e., real models), we observed a well-separation between the random and real models for the panel of ACPP + CLU (Fig. 2b) and with the addition of urinary PSA (Fig. 2c).

**Figure 3:**
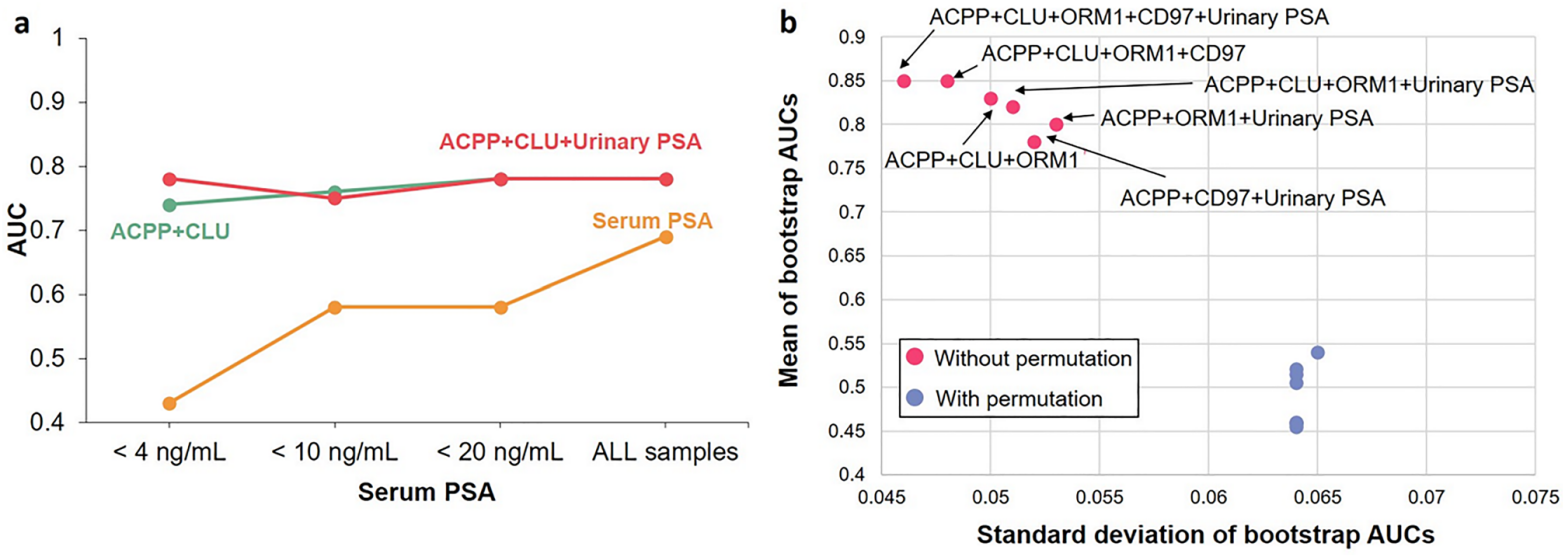
Evaluation of urinary marker panels. (**a**) Performance of the urinary marker panels composed of ACPP + CLU and ACPP + CLU + urinary PSA at different serum PSA levels in comparison to serum PSA. (**b**) Other multi-urinary marker panels with good performance compared to random models from label permutation.

Since the aforementioned multi-signature panels showed promising results, we further evaluated the performance of these urinary marker panels at different serum PSA levels (Fig. 3a and Table S5). The ability of serum PSA to distinguish aggressive PCa and non-aggressive PCa decreased along with serum PSA concentration. On the contrary, the urinary marker panels still maintained the discrimination power towards aggressive PCa detection, with AUCs ranged between 0.74 and 0.78 for the two-signature panel, and ranged from 0.75 to 0.78 for the three-signature panel. Thus, the urinary marker panels composed of ACPP, CLU, and/or urinary PSA may serve as the supplements to the serum PSA test.

Furthermore, we also evaluated different combinations of the urinary glycopeptides and/or urinary PSA into multi-signature panels with up to five candidate markers. We observed improvement in the overall performance in detecting aggressive PCa using multi-signature panels (Fig. 3b and Table S6). The AUCs of the multi-signature panels from the real models were clearly separated from the AUCs calculated from the random models, indicating the performances of these panels were statistically meaningful.

In summary, urinary PSA can be either an individual marker or in combination with other urinary glycopeptides, while urinary marker panels composed of multiple glycopeptides also demonstrated the potential in aggressive PCa detection.

### Evaluation on urinary marker panels using an independent cohort

The validation cohort contained a total of 156 urine samples, which was composed of 13 GG1, 57 GG2, 46 GG3, and 40 GG4. Since our predictive models were built using urine samples of GG1 and Grade Group ≥ 4, therefore, we first examined the performance of the panels only using urine samples of GG1 and GG4 from the validation cohort. We found that ACPP and urinary PSA still showed good performance in distinguishing GG1 and GG4 with AUCs of 0.74 and 0.78, respectively. Five other multi-marker panels demonstrated moderate performance (Table 1).

**Table 1:** Performance of urinary PSA and MS-based urinary marker panels with/without urinary PSA in the independent cohort.

Panel	AUC (95% CI)
**GG1 versus GG4**	
ACPP	0.74 (0.60, 0.88)
Urinary PSA	0.78 (0.65, 0.92)
ACPP + CLU + Urinary PSA	0.71 (0.55, 0.88)
ACPP + ORM1 + Urinary PSA	0.74 (0.60, 0.88)
ACPP + CD97 + Urinary PSA	0.79 (0.66, 0.93)
ACPP + CLU + ORM1 + CD97 + Urinary PSA	0.72 (0.57, 0.88)
**GG1 versus GG2**	
Urinary PSA	0.65 (0.51, 0.80)
ACPP + ORM1 + Urinary PSA	0.68 (0.54, 0.83)
**GG1 versus GG3**	
Urinary PSA	0.75 (0.62, 0.88)
ACPP + ORM1 + Urinary PSA	0.71 (0.57, 0.85)
ACPP + CLU + Urinary PSA	0.7 (0.54, 0.85)
**GG1 versus GG2 and above**	
Urinary PSA	0.72 (0.61, 0.84)
ACPP + CLU + Urinary PSA	0.64 (0.49, 0.79)
ACPP + ORM1 + Urinary PSA	0.71 (0.58, 0.83)
**GG1 versus GG3 and above**	
Urinary PSA	0.77 (0.65, 0.88)
ACPP + CLU + Urinary PSA	0.7 (0.54, 0.85)
ACPP + ORM1 + Urinary PSA	0.72 (0.59, 0.85)
ACPP + CD97 + Urinary PSA	0.71 (0.57, 0.84)

PCa patients with either GG2 or GG3 are all considered as in the intermediate risk. However, a PCa tumor classified as GG2 (Gleason score = 3 + 4) contains more pattern 3 and a small portion of pattern 4 which may hamper the differentiation between GG1 (Gleason score = 3 + 3) and GG2 as well as between GG1 and ≥ GG2. Therefore, we examined the performance of the panels in separating GG1 from GG2 and above as well as GG1 from GG3 and above. We found urinary PSA and a panel composed of ACPP, ORM1, and urinary PSA showing promising results in detecting aggressive PCa with Grade Group ≥ 2 (Table 1). Moreover, urinary PSA had good performance (AUC ≥ 0.75) in distinguishing GG1 and ≥ GG3 along with three other urinary marker panels (Table 1). Collectively, novel panels of candidate biomarkers for aggressive PCa were discovered showing promising results as further evaluated using an independent validation cohort.

### Performance of PRM assays in combination with urinary PSA

**Figure 4:**
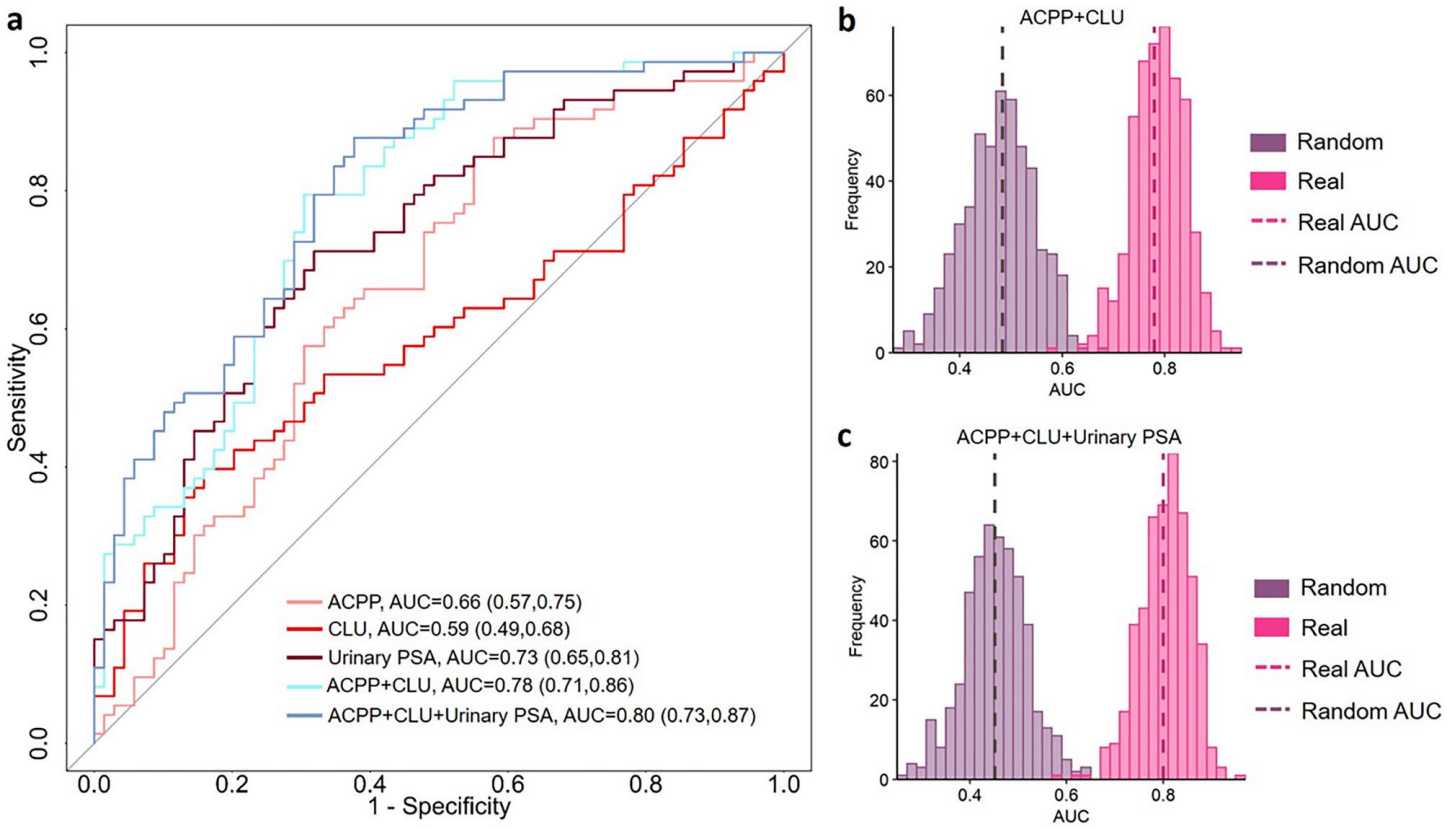
Urinary marker panels composed of PRM assays of the urinary glycopeptides from ACPP, CLU, and/or urinary PSA. (**a**) ROC analysis of CLU, ACPP, urinary PSA, and multi-signature panels. (**b**) and (**c**) Comparison between real models and randomly generated models from label permutation based on bootstrap resampling.

To evaluate the clinical utility of the candidate glycopeptides and facilitate the translation of the MS-based candidate biomarkers to routine clinical implementation in future, we developed easily extendable PRM quantitative assays for ACPP and CLU since promising results were found for aggressive PCa detection based on quantitative DIA analysis^23^. The performance of the PRM assays were first evaluated along with urinary PSA using the Cohort 1 (Table S7). An improvement in differentiating aggressive and non-aggressive PCa was observed by using a panel composed of ACPP and CLU (AUC = 0.78) compared to individual candidate markers (Fig. 4a). An AUC of 0.8 was achieved when combining ACPP, CLU and urinary PSA. We also generated and analyzed 500 random models in comparison to the real models. The random models generated median AUCs of 0.48 and 0.47 for the panel of ACPP + CLU and the panel of ACPP + CLU + urinary PSA, respectively (Fig. 4b, c), which were much lower and clearly separated from the real models.

To validate the performance of the PRM assays, we used the aforementioned independent cohort (Table 2). We found the PRM assay of the urinary glycopeptide from ACPP still maintained its ability in differentiating GG1 from GG4. The multi-marker panels also showed moderate performance in the group comparisons of (1) GG1 versus GG4, (2) GG1 versus GG3, (3) GG1 versus GG2 and above, and (4) GG1 versus GG3 and above.

**Table 2:** Performance of PRM-based urinary marker panels with/without urinary PSA in the independent cohort.

Panel	GG1 versus GG4	GG1 versus GG2	GG1 versus GG3	GG1 versus GG2 and above	GG1 versus GG3 and above
	AUC (95% CI)	AUC (95% CI)	AUC (95% CI)	AUC (95% CI)	AUC (95% CI)
ACPP	0.71 (0.54, 0.87)	0.61 (0.45, 0.78)	0.66 (0.49, 0.83)	0.66 (0.50, 0.81)	0.68 (0.53, 0.84)
ACPP + CLU	0.65 (0.48, 0.82)	0.55 (0.38, 0.71)	0.68 (0.50, 0.85)	0.62 (0.47, 0.78)	0.68 (0.52, 0.84)
ACPP + CLU + Urinary PSA	0.65 (0.48, 0.82)	0.54 (0.38, 0.70)	0.71 (0.54, 0.88)	0.62 (0.48, 0.76)	0.68 (0.53, 0.84)

In summary, the reported results elucidated that the PRM assays were successfully developed for urinary glycopeptides. The PRM assays were applicable to the quantitative analysis of targeted peptides from real clinical specimens as well as combining with urinary PSA to gain improved discrimination power.

## Discussion

PCa patients with biopsy Grade Group 1 are considered as low-risk patients^24–26^. The progression of PCa can be very slow, thus, low-risk patients may not require immediate treatment. However, monitoring the progression of the disease usually requires biopsy periodically, which can be harmful for the patients. Therefore, identifying noninvasive biomarkers has a significant clinical value. Urine is a great source for finding noninvasive biomarkers associated with PCa. The FDA-approved prostate cancer antigen-3 (PCA3), a urine-derived long noncoding RNA biomarker, can assist the decision making for repeated biopsies with reported AUCs ranged from 0.64 to 0.76^27,28^. Other urine-based genomic biomarker panels have also shown the prognostics values for PCa, including panels composed of multiple gene probes (e.g., PUR), exome (e.g., ExoDx), DNA methylation (e.g., epiCaPture), or mRNA (e.g., SelectMDx)^11,28–35^. Urine-derived proteomic biomarkers have been investigated as well^11,36^. Nevertheless, there is still a need for noninvasive biomarkers to improve the clinical performance for detecting aggressive PCa effectively. Therefore, identifying noninvasive urinary biomarkers that can differentiate aggressive PCa from non-aggressive PCa is essential to fulfill the unmet clinical needs. Previously, we utilized urine samples from low-risk (GG1) and high-risk (≥ GG4) PCa patients and discovered several urinary glycopeptides with potentials in detecting aggressive PCa. In the current study, we focused on two aspects, (1) the potential prognostic value of urinary PSA and (2) determine the clinical utility of urinary marker panels consisted of previously identified urinary glycopeptides and urinary PSA in various combinations for distinguishing aggressive PCa (GG2 and above) from non-aggressive (GG1) since GG2 and GG3 were not included in our previous work.

We used the glycoproteomic analysis approach to analyze urine samples quantitatively and built predict models from GG1 and ≥ GG4 patients for urinary PSA and glycopeptides from ACPP, CLU, ORM1, and CD97^13^. ACPP is a prostate specific protein correlates with the activation of MAPK signaling, which can result in PCa progression and androgen independent growth of PCa cells^37,38^. We previously discovered ACPP as a promising urinary marker while combining with CLU could enhance the discrimination power of aggressive PCa. In this study, we found the addition of urinary PSA moderately improve the overall performance (Fig. 2a). Moreover, serum PSA test is a common tool in diagnosing PCa, where an elevated serum PSA level is usually found in patients with aggressive PCa. However, based on the cohort used in this study, the performance of serum PSA for aggressive PCa detection dropped when serum PSA level < 20 ng/mL, whereas the urinary marker panel composed of ACPP, CLU, and/or urinary PSA showed consist performance (Fig. 3a). As the sensitivity was fixed at 95%, improvement in specificity was observed in multi-signature panels compared to individual markers (Tables S4, S6). By developing the PRM assays, we can facilitate the translation of MS-based candidate markers into routine clinical implementation. Similarly, the PRM assays of the glycopeptides of ACPP and CLU demonstrated the ability to separate aggressive and non-aggressive groups as a two-signature panel as well as a three-signature panel by including urinary PSA (Fig. 4a).

Besides urinary PSA and urinary glycopeptides from ACPP and CLU, we also investigated the addition of urinary glycopeptides from ORM1 and CD97. ORM1 is involved in androgen receptor signaling pathway and CD97 is associated with PCa cell invasion that both showed elevated expression in aggressive PCa^13,39–41^. By including ORM1 and CD97, the overall performance was improved (Fig. 3b). Two panels, ACPP + CLU + ORM1 + CD97 and ACPP + CLU + ORM1 + CD97 + urinary PSA, achieved highest averaged AUCs from bootstrap resampling compared to the other multi-signature panels. Of note, none of the aforementioned outcome was a random observation based on label permutation.

The urinary marker panels (Figs. 2, 3, 4) were further evaluated using an independent cohort, which composed of urine samples from patients with GG1 to GG4. Since Cohort 1 contained only GG1 and GG4 and above, therefore, we first validated the panels using only GG1 and GG4 from the independent cohort. Gleason score 7 is also referred to as aggressive PCa because the patients are at intermediate risk of cancer progression^42^. However, Gleason score 7 tumors can be either GG2 (Gleason score = 3 + 4) or GG3 (Gleason score = 4 + 3). The GG2 and GG3 tumors are not equivalent to each other and GG3 is more aggressive than GG2^42,43^. Thus, we had validation groups of GG1 versus GG2 and above and GG1 versus GG3 and above in addition to the GG1 versus GG4. Majority of the urinary marker panels (e.g., ACPP + CLU + urinary PSA and ACPP + CD97 + urinary PSA) still maintained the ability to distinguish GG1 from GG2 and above (Tables 1, 2) indicating the reliability of the urinary marker panels for aggressive PCa detection.

In conclusion, the current study highlights the urinary marker panels consisted of various combination of four urinary glycoproteins and urinary PSA for aggressive PCa detection, which were not explored in our previous works. Furthermore, our results suggest the feasibility of applying the DIA-MS-based and PRM assays into clinical applications in future.

## Supplementary Information

Supplementary Information.

## Data Availability

All data generated or analyzed during this study are included in this published article (and its Supplementary Information files).
